# Divergent calcium signaling in RBCs from *Tropidurus torquatus *(Squamata – Tropiduridae) strengthen classification in lizard evolution

**DOI:** 10.1186/1472-6793-7-7

**Published:** 2007-08-23

**Authors:** Flávio H Beraldo, Célia RS Garcia

**Affiliations:** 1 Universidade de São Paulo, Instituto de Ciências Biomédicas, Departamento de Parasitologia, São Paulo, Brazil; 2 Universidade de São Paulo, Instituto de Biociências, Departamento de Fisiologia, São Paulo, Brazil

## Abstract

**Background:**

We have previously reported that a Teiid lizard red blood cells (RBCs) such as *Ameiva ameiva *and *Tupinambis merianae *controls intracellular calcium levels by displaying multiple mechanisms. In these cells, calcium stores could be discharged not only by: thapsigargin, but also by the Na^+^/H^+ ^ionophore monensin, K^+^/H^+ ^ionophore nigericin and the H^+ ^pump inhibitor bafilomycin as well as ionomycin. Moreover, these lizards possess a P2Y-type purinoceptors that mobilize Ca^2+ ^from intracellular stores upon ATP addition.

**Results:**

Here we report, that RBCs from the tropidurid lizard *Tropidurus torquatus *store Ca^2+ ^in endoplasmic reticulum (ER) pool but unlike in the referred Teiidae, these cells do not store calcium in monensin-nigericin sensitive pools. Moreover, mitochondria from *T. torquatus *RBCs accumulate Ca^2+^. Addition of ATP to a calcium-free medium does not increase the [Ca^2+^]_c _levels, however in a calcium medium we observe an increase in cytosolic calcium. This is an indication that purinergic receptors in these cells are P2X-like.

**Conclusion:**

*T. torquatus *RBCs present different mechanisms from Teiid lizard red blood cells (RBCs), for controlling its intracellular calcium levels. At *T. torquatus *the ion is only stored at endoplasmic reticulum and mitochondria. Moreover activation of purinergic receptor, P2X type, was able to induce an influx of calcium from extracelullar medium. These studies contribute to the understanding of the evolution of calcium homeostasis and signaling in nucleated RBCs.

## Background

Calcium is an important intracellular messenger involved in most physiological process from protozoa [1-5] to vertebrates [6,7]. In nucleated RBCs Ca^2+ ^regulates several cell functions including membrane permeability [8] and cell differentiation [9,10]. The mechanisms of Ca^2+ ^homeostasis involve the extrusion of this ion by a Ca^2+-^ATPase on the plasma membrane and storage within intracellular organelles. Most, if not all, intracellular organelles are capable of storing Ca^2+ ^including the ER, acidic pools, and mitochondria among others [11,12]. Calcium uptake into organelles proceeds against its chemical gradient and requires energy to operate. In the ER there is a specialized Ca^2+^-ATPase (SERCA) [13]. In mitochondria the energy is provided by the membrane potential [14]. The mitochondria have the capacity to store high concentrations of Ca^2+^, not only by ATP hydrolysis but, mainly, by energy derived from substrate oxidation.

Present in the immature stage of mammalian erythrocytes, birds, amphibians, fishes and reptiles, nucleated RBCs differ from mature mammalian erythrocytes by the presence of a nucleus and organelles. Despite this, few studies of the mechanisms of Ca^2+ ^homeostasis in nucleated RBCs have been performed [15,16]. By studying signal transduction pathways in lizard RBCs from different families, we have been able to infer phylogenetic aspects and mechanisms for these cells to maintain its Ca^2+ ^homeostasis.

The squamates are divided in two large groups – Scleroglossa and Iguania. The first assembles the snakes, amphisbaenas, and a diverse array of lizard families, among them the Teiidae, while the last comprise a less diverse assort of lizard families which includes the Tropiduridae. We have previously shown that RBCs from the teiid lizards *Ameiva ameiva *and *Tupinambis merianae *(Scleroglossa) display two distinct types of intracellular compartments involved in calcium homeostasis. These cells display the P2Y type purinoceptors that are probably coupled to G protein and promote an increase of [Ca^2+^]_c _through the InsP_3 _pathway [17-20]. Here we show that RBCs of the tropidurid *Tropidurus torquatus *(Iguania) store calcium in the classical compartment ER and in mitochondria. Differently from the Teiidae studied, there is a P2X purinoceptor in *Tropidurus torquatus *that promotes an increase of [Ca^2+^]_c _by influx of Ca^2+ ^from extracellular medium.

## Results

### Intracellular Ca^2+ ^stores in *T. torquatus *RBCs

**Figure 1:**
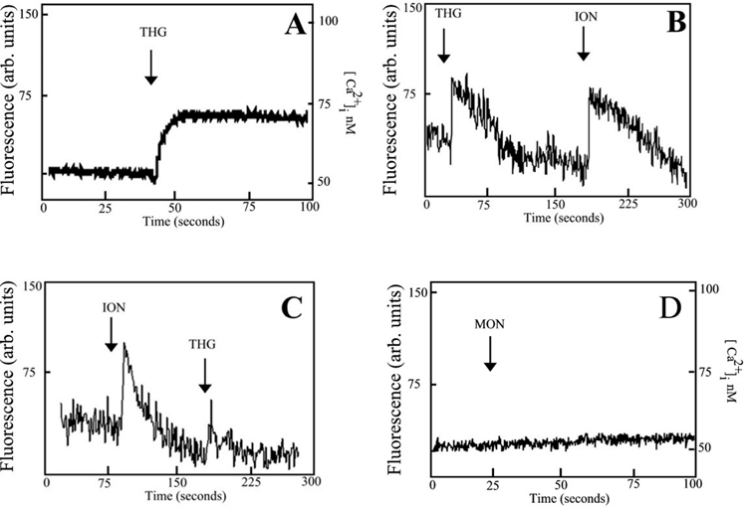
Ca^2+ ^mobilization in Fluo-3-labelled RBCs of the lizard *T. torquatus*: A) RBCs incubated in calcium medium (1 mM CaCl_2_) were treated where indicated with the SERCA inhibitor, thapsigargin (5 μM). as in A, but CaCl_2 _was omitted and the medium was supplemented with 15 mM EGTA THG (5 μM) was add before addition of Ca^2+ ^ionophore ionomycin (10 μM). C) Effect of THG (5 μM) in RBC pre incubated with ionomycin (10 μM). D) Monensin (25 μM) was not able to induce an intracellular Ca^2+ ^increase.

By using spectrofluorimetry and loading RBCs from *Tropidurus torquatus *with Fluo-3 AM, we characterized the intracellular pools involved in calcium homeostasis within these cells. We found [Ca^2+^]_c _in the RBCs of *Tropidurus torquatus *around 51,2 ± 1,7 nM (n = 18). In order to verify if *T. torquatus *RBCs contain intracellular Ca^2+ ^stores compartments, the cells were treated with the tumor promoter thapsigargin, known to specifically inhibit the Ca^2+ ^ATPase from the sarco-endoplasmic reticulum (SERCA) [21]. Fig 1A shows that the addition of thapsigargin (5 μM) resulted in a sustained increase of cytosolic Ca^2+ ^([Ca^2+^]_c_) of 18.1 ± 2.1 nM (n = 6) when the cells were incubated in Ca^2+ ^medium (1 mM). Addition of thapsigargin in Ca^2+^-free medium (supplemented with 15 mM EGTA) resulted in an increase of fluorescence in RBCs of *T. torquatus *loaded with Fluo-3 AM. Subsequent addition of ionomycin (10 μM) resulted in further increased fluorescence (Fig 1B) indicating the presence of other Ca^2+^-stores. If the calcium pools were depleted by ionomycin, thapsigargin does not elicit any further calcium release (Fig 1C). The increase in arbitrary fluorescence units observed on Fig 1B and 1C indicate an increase on intracellular Ca^2+ ^on Tropiduridae lizards RBCs. As these experiments were performed in a free Ca^2+ ^medium we therefore could not calculate the intracellular changes on Ca^2+.^.

Differently from Teiidae family, RBCs from Tropiduridae lizards are not sensitive to Monensine (ionophore Na^+^/H^+^) (Fig 1D) nor Nigericine (ionophore K^+^/H^+^) (data not shown).

### Purinergic receptors in *T. torquatus *RBCs

**Figure 2:**
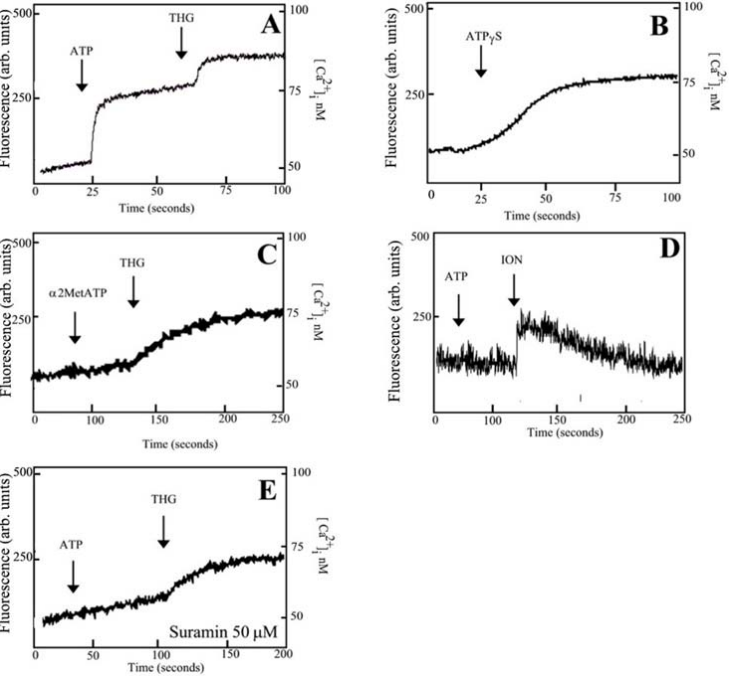
Effect of purinoceptors agonists in RBC loaded with Fluo-3 AM. A) ATP (50 μM), in Ca^2+ ^medium (1 mM CaCl_2_). B-C) Effect of a non hydrolysable analogue of ATP – ATPγS (50 μM) and a specific P2Y agonist – α2MetATP (50 μM) respectively. D) Effect of ATP (50 μM), in a free Ca^2+ ^supplemented with medium 15 mM EGTA. E) Inhibition of ATP effect by a P2 antagonist – Suramin (50 μM).

ATP triggers calcium release in the RBCs of *Ameiva ameiva *and *Tupinambis merianae *lizards. In these cells, we have characterized the G-protein coupled P2Y type receptor as ATP mobilized calcium from internal pools in a calcium-free medium. We also reported that the second messenger InsP_3 _could be the signal for calcium mobilization from internal pools within these cells. In *Tropidurus torquatus*, the addition of ATP (50 μM) promotes a sustained increase of [Ca^2+^]_c _of 26.4 ± 3.4 nM (n = 10) when the cells are incubated with Fluo-3 AM in Ca^2+ ^medium (CaCl_2 _1 mM) (Fig 2A). To verify if the purinergic receptor was a P1 (binding only adenosine) we have used a non hydrolysable ATP analogue ATPγS. In Fig 2B, we showed that ATPγS (50 μM) induced an increase in the [Ca^2+^]_c _of 33.2 ± 3.4 nM (n = 4). To observe the presence of a P2Y purinoceptors we treated the RBCs with a specific antagonist to this type of receptor. We have shown that addition of α2MetATP (50 μM) did not induce an increase of [Ca^2+^]_c _in RBCs from *T. torquatus *loaded with Fluo-3 AM (Fig 2C). When the experiments were performed in a medium without Ca^2+ ^in the presence of EGTA (15 mM) no effect was observed in fluorescence with addition of ATP (50 μM), although further addition of ionomycin (10 μM) promotes Ca^2+ ^release (Fig 2D). When we incubated the RBCs-loaded with Fluo-3 AM- with the purinergic receptors P2 type inhibitors such as Suramin (50 μM) (Fig 2E), for 30 min, no [Ca^2+^]_c _increase was observed with addition of ATP (50 μM). These results indicate that the increase of [Ca^2+^]_c_, promoted by purinergic receptors agonists, is from extracellular medium by activation of a Ca^2+^-channel in the plasma membrane, probably a P2X type.

### Role of mitochondria in Ca^2+ ^homeostasis

**Figure 3:**
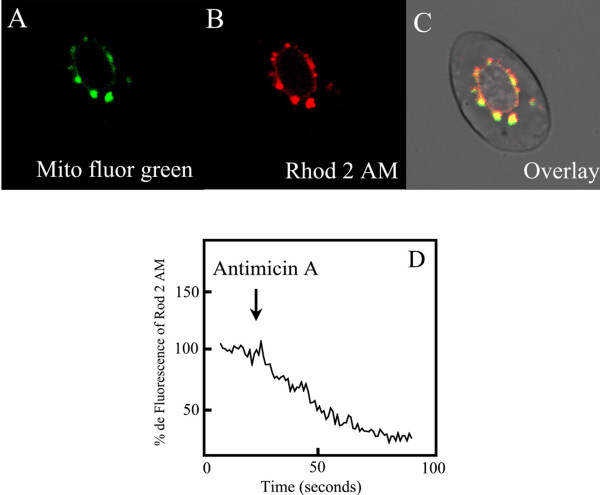
Loading mitochondrial Ca^2+ ^in RBCs from *T. torquatus*. A) Mitochondria labeling with Mito Fluor Green. B) The mitochondrial Ca^2+ ^probe, Rhod 2 AM. C) Co-localization of mitochondrial indicator with mitochondrial Ca^2+ ^indicator. D) Mitochondrial gradient was disrupted by addition of Antimycin A (5 ng/ml).

To verify the role of mitochondria on Ca^2+ ^storing in RBCs from *T. torquatus *we loaded the erythrocytes with mitochondria indicator Mito Fluor Green (5 μM) (Fig 3A) (n = 3) and with Ca^2+ ^mitochondrial indicator Rhod-2 AM (5 μM) (Fig 3B) (n = 3). Co-localization of the indicators (Fig 3C) denotes the presence of mitochondria around the nucleus. Similar to ER in *T. torquatus *RBCs mitochondria are able to store Ca^2+^. The disruption of mitochondrial potential by Antimicin A (5 ng/ml) (Fig 3D) (n = 3), FCCP (25 μM) (n = 3) and Oligomicine (0,01 ng/ml) (data not shown) denotes its role on Ca^2+ ^storage.

**Figure 4:**
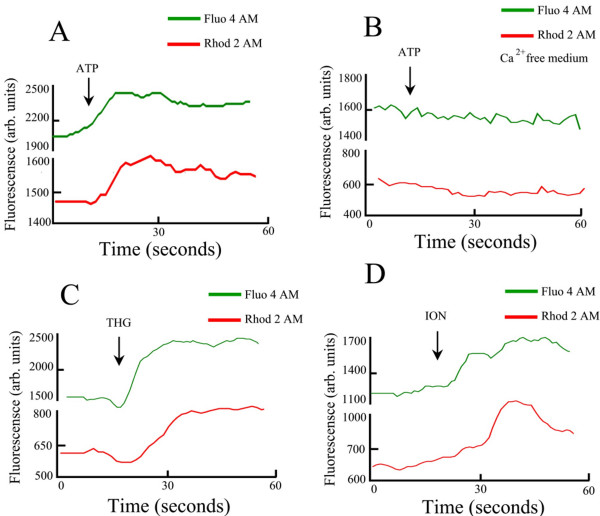
Loading of *T. torquatus *RBC with citosolic Ca^2+ ^Fluo-4 and mitochondrial Ca^2+ ^Rhod-2. A) *T. torquatus *RBCs loaded with citosolic Ca^2+ ^indicator Fluo 4 AM (Left), mitochondrial Ca^2+ ^indicator Rhod 2 AM (center) and merge of Fluo 4 and Rhod 2 (right). B) Increase of mitochondrial Ca^2+ ^[Ca^2+^]_mt _after increase of [Ca^2+^]_c _induced by ATP (50 μM) in *T. torquaturs *RBCs loaded with both Fluo 4 AM and Rhod-2 AM, in the Ca^2+ ^medium. C) In a free Ca^2+ ^medium ATP did not induce an increase of [Ca^2+^]_c _and in this case we did not observe an increase of [Ca^2+^]_mt _D and E) Similar to ATP thapsigargin (5 μM) and ionomycin (10 μM) promote an increase of [Ca^2+^]_c _with concomitant increase of mitochondrial Ca^2+^.

**Figure 5:**
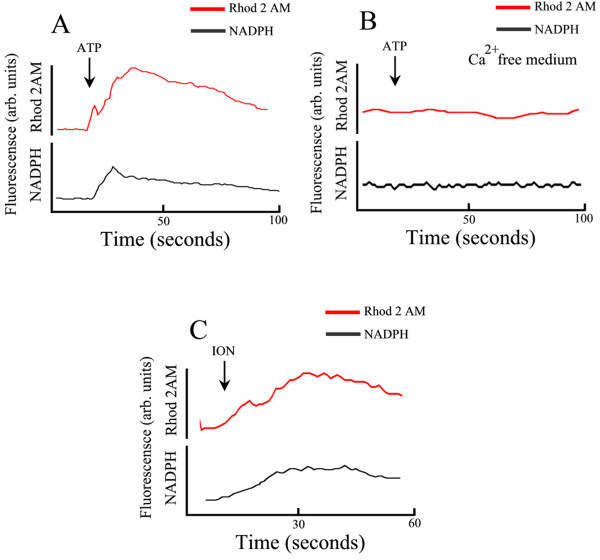
Increase of mitochondrial Ca^2+ ^promotes an increase of NADPH in *T. torquatus *RBCs loaded with mitochondrial Ca^2+ ^Rhod-2. A) Increase of mitochondrial Ca^2+ ^[Ca^2+^]_mt _and fluorescence of NADPH induced by ATP (50 μM) in *T. torquaturs *RBCs loaded with Rhod-2 AM, in the Ca^2+ ^medium. B) similar experiments in a Ca^2+ ^free medium C) Ionomycin (10 μM) promotes a increase of [Ca^2+^]_mt _with concomitant increase of NADPH fluorescence.

Mitochondrion has an important role on Ca^2+ ^homeostasis in *T. torquatus *RBCs. These cells were incubated with the citosolic Ca^2+ ^indicator Fluo 4 AM and with mitochondrial Ca^2+ ^indicator Rhod-2 AM. The addition of ATP (50 μM), as shown previously in Fig 2A, induced an increase of intracellular Ca^2+ ^by activation of a membrane purinoceptor P2X type that promotes an influx of the ion. Here we show the increase of mitochondria [Ca^2+^]_mt _after an increase of [Ca^2+^]_c _induced by ATP (50 μM) in *T. torquaturs *RBCs loaded with both Fluo 4 AM and Rhod-2 AM (in the Ca^2+ ^medium) (Fig 4A) (n = 4). In contrast to what is shown above, in a free Ca^2+ ^medium, ATP did not induce an increase of [Ca^2+^]_c _and in this case we did not observe an increase of [Ca^2+^]_mt_. (Fig 4B) (n = 4). Similar results were observed when we depleted the ER by the addition of Thapsigargin (5 μM) (Fig 4C) (n = 4) or with the ionophore ionomycin (10 μM) (Fig 4D) (n = 4). The experiments in Fig. 5 provide evidence that there is a temporal correlation between the mitochondrial Ca^2+ ^rise and the levels of NADPH. When we promoted an increase of citosolic Ca^2+ ^by ATP (Fig 5A) or ionomycin (Fig 5C) in a Ca^2+ ^medium, we observed an increase of mitochondrial Ca^2+ ^and a concomitant increase of auto fluorescence of NADPH. However in a free Ca^2+ ^medium ATP is not able to induce an increase of [Ca^2+^]_c _and in this case there is no increase of mitochondrial Ca^2+ ^or NADPH (Fig 5B) (n = 3).

## Discussion

The effect of SERCA inhibitor thapsigargin indicates that in RBCs from *T. torquatus *ER can sequester Ca^2+ ^and that blockage of the pump results in a rapid release of this ion to the cytosol. The fact of the increase of [Ca^2+^]_c_, induced by thapsigargin, in *T. torquatus *was lower than the values found for RBCs in the previously studied teiid lizards suggests that the capacity of the ER to sequester Ca^2+ ^is higher in the Teiidae, or the affinity of SERCA pump to thapsigargin is lower in *T. torquatus*. Differences on SERCA pump sensitivity to thapsigargin have been reported in different models. For example, micromolar concentrations of thapsigargin inhibit SERCAs in non-mammalian vertebrate cells of turtles [22,23] and chicken granulosa cells [24] or parasites [25,26]. Thapsigargin induced a sustained intracellular Ca^2+ ^increase in a calcium medium. Differently, in a calcium free medium a calcium spike occurs. These data indicated that, in a calcium medium, there is Ca^2+ ^mobilization from ER and an activation of influx of Ca^2+ ^from extracellular medium. This mechanism, known as "Capacitative Calcium entry" is very common in non excitable cells [27] and our previous reports have shown the presence of this mechanism on RBCs from Teeidae family [28,29].

Not only ER but almost all intracellular organelles are able to store Ca^2+ ^The diversity of Ca^2+ ^pools varies among different cell types. Here we show that endoplasmic reticulum and mitochondria are able to store Ca^2+ ^in RBCs from *T. torquatus*. We also observed that ionomicyn was able to deplete calcium stores after thapsigargin treatment. However reversing the addition do not result on calcium release from thapsigargin. This data indicates the existence of a third calcium pool in RBCs from *T. torquatus*. This could be a Golgi-type Ca^2+ ^pool – which is insensitive to thapsigargin. Another possibility was the presence of unidentified subcompartment of ER expressing an thapsigargin-insensitive Ca^2+^-ATPase [30,31].

The mitochondria, in most mammalian cells loaded with fluorescent indicators, appear disperse in the cytosol [32,33]. However, as we showed, in *T. torquatus *RBCs mitochondria is located around the nucleus, similar to other nucleated RBCs [34]. This indicates that this organelle is concentrated in a small region of the cell. In nucleated RBCs the advantage of organelles being concentrated in a small region of the cell leads to an increase of hemoglobin storage and consequently increasing the oxygen transport to tissues. Mitochondria is able to accumulate Ca^2+ ^by the increase of cytosolic Ca^2+ ^induced by influx of Ca^2+ ^from intracellular medium or by mobilization from intracellular stores in several models [35-37]. The capacity of mitochondria accumulates Ca^2+ ^at higher concentrations, in response to an agonist, was explained by Rizzuto et al (1993) [38]. The authors have demonstrated the proximity of mitochondria and ER and the presence of microdomains in the cytosol where high concentrations of Ca^2+ ^are observed. By loading of mitochondrial Ca^2+ ^with Rhod 2 AM we show that in lizards RBCs mitochondria has a fundamental role in Ca^2+ ^homeostasis. There is an increase of mitochondrial [Ca^2+^]_c _promoted by mobilization of Ca^2+ ^from ER or by influx of this ion by activation of purinergic receptors in the plasma membrane.

In squamates all types of purinergic receptors have been found. In the same organ or tissue most subtypes of receptor are present. For example, in the aorta the lizard *Agama sp *(Iguania) (type P1 and P2) [39] and the snake *Thamnophis sirtilis *(type P2X and P2Y) [40]. In nucleated RBCs the presence of purinoceptors (P2Y-coupled to G protein) have been reported in avian species [41] and lizards from Teiidae family [42]. In *T. torquatus *our results indicate the presence of a P2X (ion channel) purinergic receptor that promotes an influx of Ca^2+ ^from an extracellullar medium. We also show that P2 purinergic receptor antagonist Suramin [43] is able to block the effect of ATP in RBCs from *T. torquatus*. The presence of other types of purinoceptors cannot be ruled out. Possibly there are other signaling pathways involved in the signal transduction in the lizard's RBCs. For example, RBCs from *T. torquatus *could show a coupled G-protein purinoceptor that promotes an increase or decrease of other second messengers, such as cAMP or cGMP.

Probably these mechanisms appeared separately in the evolution of these lizard's families.

## Conclusion

In this work we founded that *T. torquatus *RBCs do not store calcium at acidic pools and display different mechanisms for controlling intracellular calcium levels when compared with RBCs from the *Teiid *family. Interestingly, the purinergic receptors present in these cells was found to be a P2X type. These receptors were activated in a medium containing extracellular calcium.

These studies open new perspectives to comprehend how calcium homeostasis and signaling in nucleated RBCs evolved.

## Methods

### Lizards

Specimens of *Tropidurus torquatus *were obtained at Lageado, state of Tocantins, Brazil. The blood, collected from the lizard's tail with a syringe, was centrifuged at 1500 g for 5 min and washed in phosphate-buffered isotonic saline (PBS: 7.5 mM sodium phosphate, 137 mM NaCl, pH 7.2). Leucocytes were removed from RBCs by subjecting the blood to a cellulose powder column (Whatman CF11, fibrous long Sigma, U.K.) adapted from Homewood and Neame (1976) [44]. The animals were bleeded on intervals of 3 months and after 3 bleeding the animals were sacrificed. The animals were used in accord with animal protocol approval 089/03 "Comparative studies of cell signaling in malaria parasite diseases – Brazilian College of Animal Experimentation and Biomedical Sciences Institute/USP-Ethical Committee for animal research" (22/09/2003 Meeting).

### Spectrofluorimetric determinations with the fluorescent dye Fluo-3

After the passage in the cellulose column, the cells were washed 3 times in buffer A (116 mM NaCl, 5.4 mM KCL, 0.8 mM MgSO_4_, 5.5 mM D-glucose, 1 mM CaCl_2 _and 50 mM MOPS, pH 7.2) at room temperature by centrifugation at 1500 g for 5 min and ressuspended (at 10^6 ^cells/ml^-1^) in same buffer containing 1.8 mM probenecid, an inhibitor of organic anion transport to prevent fluorochrome release and sequestration [45]. Spetrofluorimetric measurements white Fluo 3 AM were performed by using model F-4500 Hitachi spectrofluorimeter (Tokyo, Japan) with excitation at 505 nm and emission at 530 nm. The calibration curves for Ca^2+ ^concentration were calculated using the Ca^2+ ^software F-4500 Intracellular Cation Measurement System – Version 1.02 Copyright ^© ^Hitachi, Ltd., 1994–1995 with takes into account that; [Ca^2+^] = K_d _(F-F_min_)/(F_max_-F) where the K_d _utilized for Fluo 3 was 390 μM, F is the measured fluorescence intensity in the condition of the experiment, F_max _the fluorescence in the presence of digitonin and F_min _the fluorescence in the presence of 8 mM of EGTA. Unless otherwise specified all experiments were performed at 37°C. All the experimental conditions and the range of drug concentration were previously standardized by teiid lizards as described by Beraldo et al. 2001 and 2002 [46,47].

### Mitochondrial calcium in *T. torquatus *RBCs

In these experiments we used the mitochondrial probe Mitofluor Green and the mitochondrial Ca^2+ ^indicator Rhod-2 AM. *T. torquatus *RBCs (at 10^6 ^cells/ml^-1^) are resuspended in buffer A where Mitofluor Green (5 μM) and Rhod-2 AM (5 μM) are added. The RBCs are incubated at 37°C by 30 min. After incubation, the RBCs are washed three times in buffer A to remove the extracellular dye. The cells are observed in confocal microscopy LSM-510 Zeiss using wavelengths: Mitofluor Green EX = 490/EM = 516 and Rhod-AM EX = 545/EM = 560–615 nm. Samples were observed using slides that were previously incubated for 1 hour with polylisine in PBS 2 mg/ml and washed after incubation.

### Simultaneous recording of Fluo-4 and Rhod

Lizard RBCs are ressuspended in 1 ml of buffer A where 10 μM Fluo-4 AM and 40 μM of probenecid were added. RBCs are incubated for 60 minutes at 37°C with Fluo-4 AM. After that, 5 μM of Rhod-2 AM were added and the RBCs were incubated for 30 minutes at 37°C. Finally, RBCs were washed three times with buffer A to remove the extracellular dyes. The experiments were performed in confocal microscopy in plates previously incubated with polylisine using the wavelengths (EX = 488/EM = 550) Fluo 4 AM and (EX = 545/EM = 560–615 nm) for Rhod-2 AM.

### Simultaneous recording of Rhod and NADPH

The changes in mitochondrial Ca^2+ ^elicit an increase of activation of mitochondrial dehidrogenases, which can be detected by measuring the level of NADPH fluorescence [18]. After the passage in the cellulose column, the lizard's RBCs were washed 3 times in buffer A (116 mM NaCl, 5.4 mM KCL, 0.8 mM MgSO_4_, 5.5 mM D-glucose, 1 mM CaCl_2 _and 50 mM MOPS, pH 7.2) at room temperature by centrifugation at 1500 g for 5 min and resuspended (at 10^6 ^cells/ml^-1^) in the same buffer. After incubation with 5 μM de Rhod-2 AM the cells were washed three times with buffer A to remove the extracellular dyes. The experiments were performed in spectrofluorimeter Shimadzu using the wavelengths EX = 540/EM = 570 for Rhod 2 AM and EX = 350/EM = 460 nm for NADPH auto fluorescence.

## Authors' contributions

FHB and CRSG designed the experiments. FHB carried out the experiments with RBCs. FHB and CRSG wrote the manuscript and designed the figures. All the authors read and approved the manuscript.
